# Home-Processed Red Beetroot (*Beta vulgaris* L.) Products: Changes in Antioxidant Properties and Bioaccessibility

**DOI:** 10.3390/ijms17060858

**Published:** 2016-06-01

**Authors:** Burcu Guldiken, Gamze Toydemir, Kubra Nur Memis, Sena Okur, Dilek Boyacioglu, Esra Capanoglu

**Affiliations:** 1 Department of Food Engineering, Faculty of Chemical & Metallurgical Engineering, Istanbul Technical University, 34469 Maslak, Istanbul, Turkey; guldikenb@itu.edu.tr (B.G.); memis@itu.edu.tr (K.N.M.); okursen@itu.edu.tr (S.O.); boyaci@itu.edu.tr (D.B.); 2 Department of Food Engineering, Faculty of Engineering & Architecture, Okan University, 34959 Akfirat-Tuzla, Istanbul, Turkey; gamze.toydemir@okan.edu.tr

**Keywords:** red beetroot, *Beta vulgaris* L., home-processing, antioxidant, betalain, *in vitro* digestion, bioaccessibility

## Abstract

In this study, the effects of home-processing on the antioxidant properties and *in vitro* bioaccessibility of red beetroot bioactives were investigated. For this purpose, fresh red beetroot and six different home-processed red beetroot products—including boiled, oven-dried, pickled, pureed, juice-processed, and jam-processed—were analyzed and compared for their total phenolic (TP) and total flavonoid (TF) contents, total antioxidant capacities (TAC), and individual anthocyanin contents. In addition, bioaccessibility of red beetroot antioxidants was determined using an *in vitro* simulated gastrointestinal digestion method. Dried, pureed, and fresh red beetroot samples had the highest TP, TF, and TAC values, which were 347 ± 23 mg gallic acid equivalent (GAE)/100 g, 289 ± 53 mg rutin equivalent (RE)/100 g, 3889 ± 982 mg trolox equivalent antioxidant capacity (TEAC)/100 g, respectively. The *in vitro* digestion method revealed the highest recovery for TP (16%) and TAC (1.3%) in jam. This study provides comparative data to evaluate the effects of various home-processing techniques on antioxidant potential of red beetroot products.

## 1. Introduction

Nowadays, a new diet-health paradigm highlighted the positive aspects of diet, which has led to the nutritional studies examining foods for their protective and disease preventing potential [1]. Accordingly, fruits and vegetables became more important in human diet as “functional foods” which are capable of preventing or postponing the onset of several chronic diseases as a result of their phytochemical contents [1,2]. The Five-a-Day program became evident in 1989 with the report by National Academy of Sciences, suggesting the consumption of fruit and vegetables daily (five or more times) to decrease the risk of certain chronic diseases [3]. Therefore, people tend to consume fruit- and vegetable-based products with high levels of bioactive phytonutrients. Within this context, red beetroot (*Beta vulgaris* L.) is preferred as a rich source of betacyanin, having the group of reddish to violet betalain pigments which are majorly composed of betanins and isobetanins [4,5]. Betalains and phenolic compounds that exist in red beetroot have been reported to increase the resistance of low density lipoproteins (LDL) to oxidation and to prevent cancer and cardiovascular diseases by reducing the oxidative effect of free radicals on lipids [6,7,8]. It was also reported that beetroot juice may help to lower blood pressure and protect liver damage when included in the diet [9,10]. Furthermore, consumption of beetroot-added bread had a positive effect on lowering blood pressure [11].

Various processing methods applied to food materials may have significant effects on their antioxidant potential and bioaccessibility of included phytochemicals. Related studies on red beetroot which investigated the effects of juice-processing [12], vacuum-microwave drying [13], irradiation [14], and fermentation [15] processes on antioxidant compounds, reported an enhancing impact on the antioxidant capacity and pigment stabilization. On the other hand, high temperatures and long drying times used in hot air drying [16] were determined to decrease color retention in red beetroot [17]. In addition, convective-drying and vacuum-drying techniques were indicated to cause a reduction on phenolic compounds and antioxidant capacity [18]. Several food-processing methods, such as grinding, fermentation, and/or mild heating may enhance bioaccessibility of bioactive phytonutrients through break down of the cell walls of plant tissues or the nutrient-matrix complexes, or conversion into more active molecular structures [19].

Based on the above considerations, the purpose of this study was to investigate the effects of various traditional home-processing techniques (pickling, drying, juicing, pureeing, and jam-making) on red beetroot antioxidants and to compare them with the fresh product. For this purpose, total phenolic (TP) and total flavonoid (TF) contents, total antioxidant capacities (TAC), and individual anthocyanin contents of fresh and processed red beetroot products were analyzed. Furthermore, *in vitro* simulated gastrointestinal digestion method was performed to determine the changes in the bioaccessibility of red beetroot antioxidants with processing.

## 2. Results and Discussion

### 2.1. Moisture Content

**Figure 1:**
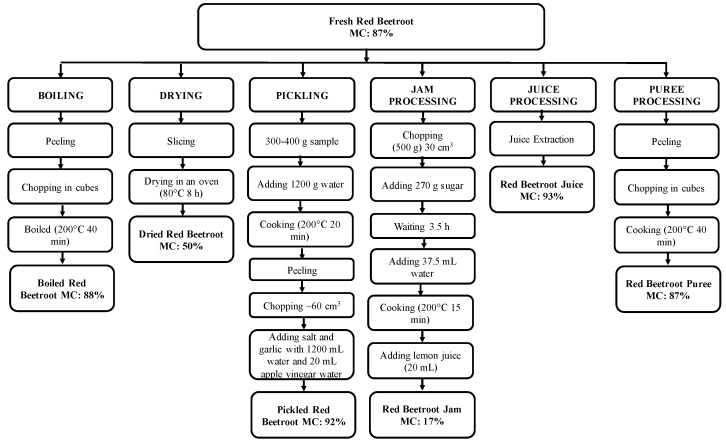
Home-processing methods of boiled, oven-dried, pickled, pureed, juice-processed, and jam-processed red beetroot samples, that samples moisture content (MC) is presented.

Moisture content results of fresh and processed red beetroot samples were given in Figure 1. While the highest moisture contents were measured for red beetroot juice (93%) and pickled red beetroot (92%) samples; red beetroot jam had the lowest moisture content value (17%). Because of the fact that dried red beetroot samples were not totally dried as in the case of industrial drying process, the moisture content measured for dried samples (50%) was higher than the jam-processed red beetroot (17%) for which the moisture content could be affected by the added-sugar and by the cooking process applied.

### 2.2. Total Phenolic Content

The results obtained for TP contents of fresh and six different home-processed red beetroot products indicated the highest value in dried sample which was measured as 347 ± 24 mg gallic acid equivalent (GAE)/100 g sample on wet basis (Table 1). Drying process led to 36% higher TP contents in dried red beetroot compared to the fresh sample. This could be linked to the higher dry weight value in dried sample (50%, wet basis) in comparison to the fresh red beet root (13%, wet basis), which could give rise to over-representation of the measured values on fresh weight basis. On dry basis, TP contents of fresh samples was determined as 1964 ± 369 mg GAE/100 g sample and drying caused 65% lower TP values. In a similar manner, drying of red grape pomace peels at 100 and 140 °C resulted in 18.6% and 32.6% decreases in the contents of total extractable polyphenols, respectively [20].

In addition, the difference of TP values of juice and jam samples were found to be statistically insignificant and was measured on a wet basis as 225 ± 15 mg GAE/100 g sample, and 231 ± 36 mg GAE/100 g sample, respectively. However, on dry weight basis, TP values after juice processing was found as 3217 ± 214 that expresses 64% increase in comparison to the fresh sample. This increase in TP values may be related to the increases in free (extractable) flavanols [21,22]. On the other hand, TP values after jam processing were found to be 86% lower than the fresh sample (278 ± 44 mg GAE/100 g sample) on dry basis. TP values of jam samples may be underestimated due to the total solid content resulted from added sugar.

The home-processing steps applied for boiled red beetroot (Figure 1) did not have significant effects on TP values when compared to the fresh sample (Table 1) (*p* < 0.05). Furthermore, when the TP contents of boiled (238 ± 15 mg GAE/100 g sample, wet basis) and pureed (236 ± 18 mg GAE/100 g sample, wet basis) samples, which had similar processing steps except for the last step of puree making in pureed sample, were compared, it could be indicated that the mechanical stress during puree making did not lead to a significant change on the TP content (*p* < 0.05). Indeed, boiled and pureed samples had similar moisture contents (12%–13%) to fresh samples, thus on a dry basis there was no significant difference between these products (*p* < 0.05).

On wet basis, the lowest TP content was determined for pickled red beetroot, having a 25% lower TP content in comparison to the fresh sample (Table 1). Based on this, it could be stated that fermentation process did not promote the total phenolic content of red beetroots. In accordance with our findings, pickling of potherb mustard was also reported to result with decreases in TP content, indicating the tendency of phenolic degradation [23]. This result can be explained by the degradation of phenolics which is mainly caused by the enzyme polyphenol oxidase, and can be controlled during the pickling process by the inhibition of this enzyme through the application of proper salt content and acidity [23,24]. On the other hand, when evaluated on dry basis, TP content of pickled red beetroot was found as 2400 ± 620 mg/100 g sample which indicates 22% increase in TP content when compared to the fresh sample. Similarly, TP contents of black soybeans and soymilk were reported to increase significantly via fermentation [20,21,22,23,24,25]. Enzymes produced by starter microorganisms (*i.e.*, β-glucosidase) during fermentation may catalyze the release of phenolics [26] which are usually found in plant materials as conjugated with sugar and glycosides via hydroxyl groups [27], leading to increased contents of those compounds in fermented products [23,24].

### 2.3. Total Flavonoid Content

The TF contents of fresh and home-processed red beetroot samples were given in Table 1. The puree processing was found to increase the TF content (290 ± 53 mg RE/100 g sample, wet basis) by 12% when compared to the TF content of fresh sample (260 ± 13 mg RE/100 g sample, wet basis). Similarly, puree processing was reported to provide up to a two-fold increase in TF content of fresh tomatoes on a fresh weight basis [28]. On the other hand, the boiling process, having similar processing parameters with puree making process except for the last pureeing step, provides similar effects to puree processing (Table 1) on a wet basis. However, boiling and puree processing did not provide a statistically significant difference on the TF contents of the fresh sample (*p* < 0.05). The results were in parallel due to similar dry weight contents of fresh (13%), pureed (13%), and boiled (12%) samples. However, TF content of red beetroot juice, in which a mechanical stress was also included during juice extraction step, induced 52% lower TF content on wet basis and 10% lower TF content on dry basis than that of the fresh sample. These three processes of boiling, puree-, and juice-processing included thermal and/or mechanical stresses. While juice processing included a mechanical stress, puree processing contained not only a mechanical stress but also a thermal process. Although the thermal process itself did not lead to a change in the TF content in boiled samples and mechanical stress led to a two-fold decrease in the TF content of red beetroot juice sample; the thermal process combined with mechanical stress resulted in a 12% increase of TF content in red beetroot puree compared to the fresh red beetroot sample on wet basis. In a study, quercetin contents of onions and tomatoes were measured after several thermal processing steps including frying, boiling, and microwave cooking, and were reported to result in 20%–35%, 75%–80% increases, and a 65% decrease, respectively [29,30]. On the other hand, as another thermal process drying decreased the TF content by 11% on wet basis and 77% on dry basis. In addition, jam processing resulted with 45% and 91% lower TF contents on wet and dry basis, respectively.

### 2.4. Total Antioxidant Capacity

The effects of home-processing methods on TAC of red beetroots determined using DPPH (1,1-diphenyl-2-picrylhydrazyl), CUPRAC (Cupric Ion Reducing Antioxidant Capacity), ABTS (2,2-azinobis (3-ethylbenzothiazoline-6-sulfonic acid) diammonium salt), and FRAP (Ferric Reducing Antioxidant Power) assays were presented in Table 1. Drying and puree making processes did not significantly alter the TAC values on a wet basis compared to the values obtained for fresh samples as determined using four *in vitro* tests in parallel (except for the FRAP assay which indicated significantly lower TAC results in puree sample compared to the fresh sample) (*p* > 0.05). However, on a dry basis, TAC values of red beetroot decreased by 72%–76% after drying process in comparison with the fresh sample in all assays. Furthermore, in our study, boiling, pickling, jam-, and juice-processing were found to lead to significant decreases in TAC values compared to the fresh sample on wet basis (except for DPPH assay) (Table 1) (*p* < 0.05). Taking into consideration that the red beetroot jam samples represented the highest dry weight content (83%), dramatic decreases were determined only in jam processing (85%–88%) in all assays. The lowest TAC values were recorded for pickled (122 ± 29 and 66 ± 5 mg TEAC/100 g sample on wet basis in ABTS and FRAP methods, respectively) and juice-processed (110 ± 9 and 2397 ± 195 mg TEAC/100 g sample on wet basis in DPPH and CUPRAC methods, respectively) red beetroot samples (Table 1). These lower antioxidant activities determined for pickled and juice-processed samples could be linked to their relatively lower TP, as well as lower dry matter contents, compared to the other home-processed red beetroot samples. A negative effect of pickling on antioxidant activity of potherb mustard was also reported in a similar study which was also described for the losses in TP content [23].

### 2.5. Individual Red Beetroot Antioxidants

**Figure 2:**
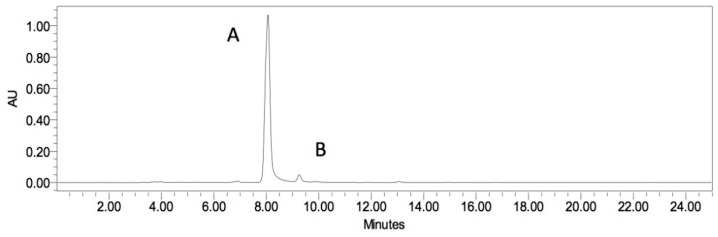
The representative HPLC chromatogram obtained for fresh red beetroot sample at 520 nm. Peaks: A = betanin; B = isobetanin.

A representative HPLC chromatogram of fresh red beetroot was shown in Figure 2. The two major peaks obtained were identified as betanin (peak A in Figure 2) and isobetanin (peak B in Figure 2), as also reported by Stuppner and Egger [31].

**Figure 3:**
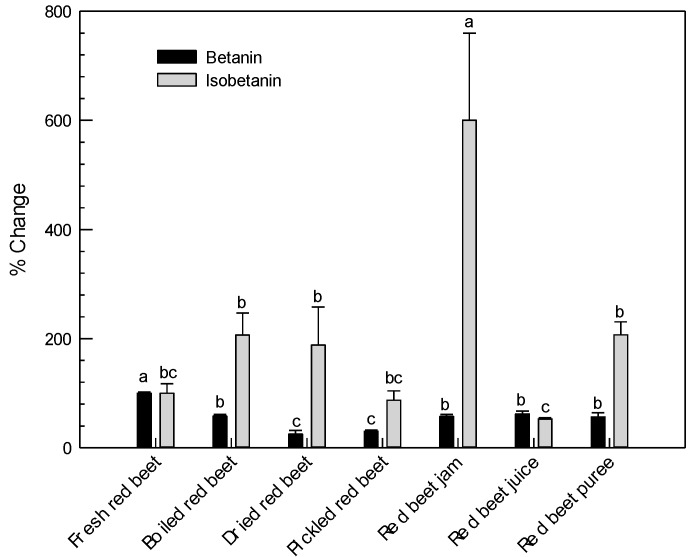
Percentage of change in betanin and isobetanin contents of processed red beetroot samples in reference to fresh samples. Results of fresh samples were taken 100% *. * Graph represent average values ± standard deviation of three independent extractions from each sample. Different letters represent statistically significant differences (*p* < 0.05).

The percent changes in the contents of the identified individual red beetroot antioxidants, betanin and isobetanin, during processing were shown in Figure 3 in which the initial contents determined in fresh red beetroot were represented as 100%. The betanin content was found to be decreased significantly during processing (*p* < 0.05), resulting with 75%, 70%, 43%, 43%, and 35% lower values in dried, pickled, pureed, jam, and juice samples, compared to the fresh sample, respectively. Significantly lower betanin concentrations determined in all home-processed red beetroot samples may be due to the isomerization, decarboxylation, and/or cleavage of betacyanins during processing, by heat and/or acid effect [32]. Depending on the different home-processing methods applied, the degree of reductions in the initial betanin concentration were also different. The highest percent reduction in betanin content was measured to be in dried red beetroot sample which could be linked to the low thermal stability of betalains [33]. In accordance with this hypothesis, the lowest percent decreases were measured for red beetroot juice which was the only sample not subjected to heat treatment during processing (Figure 1). Likewise, it was reported that thermal degradation of betalains in milk samples containing red beetroot powder increased with higher temperatures [34].

On the other hand, home-processed red beetroot samples (except for red beetroot juice) were found to have higher isobetanin concentrations compared to the fresh sample. Although the processed red beetroot samples, other than the red beetroot juice, had increased isobetanin contents; the highest increase was detected for red beetroot jam which had increased isobetanin concentration up to six-fold compared to the fresh sample (Figure 3). Higher dry weight content of red beetroot jam (83%) than the dry weight contents of the other processed red beetroot samples—which were 8%, 7%, 13%, 50%, and 12% for pickled, juice-processed, pureed, dried, and boiled red beetroot, respectively—could influence the over-representation of isobetanin concentration in this sample. Thermal processes applied during production of home-processed red beetroot samples (except for the juice sample), may be the reason for these higher isobetanin concentrations in heat-treated samples as a consequence of the epimerization of betanin to isobetanin [35]. This might also explain the reductions in betanin contents during processing which were accompanied by increases in isobetanin contents.

### 2.6. Processing Effects on in Vitro Bioaccessibility

The effects of *in vitro* gastrointestinal digestion on TP, TF, and TAC of fresh and processed red beetroot samples were given in Table 2, Table 3 and Table 4, respectively. After gastric digestion (PG), TP content values of all samples decreased to one-third to one eight of the initial values (Table 2). Our findings are in accordance with the data obtained by Helal *et al.* [36] who reported a 21% loss in TP content in cinnamon beverages after PG digestion substantially related to digestive enzymes. In contrast, TP content values of processed tomato products [28] and beetroot shots [37] were reported to show four- and three-fold increases after gastric digestion, respectively. After intestinal digestion, the highest recovery (calculated as “content in IN (dialyzed fraction after intestinal digestion) fraction/content in initial sample”) in TP content was found in jam samples (~16%). It was already known that Folin-Ciocalteau assay was susceptible to the possible interferences during *in vitro* digestion procedure [38], and so it may be better to consider other assays to determine the effects of home-processing on bioaccessibility of red beetroot bioactives.

Red beetroots are rich sources of phenolics, however in the small intestine the amount of compounds that are available for absorption might be much smaller. On the other hand, non-absorbed compounds in the small intestine can be converted or degraded by the colon microflora. It has been also reported that the metabolites might have a positive influence on the large intestine cells or bacteria and also be absorbed and show a biological activity away from the large intestine [39].

*In vitro* TF content in PG fraction of dried red beetroot was found to be increased by 20%. In addition, the highest recovery of TF contents after intestinal digestion was also found in dried samples (33%). On the other hand, drying process also led to the highest TF content in non-dialyzable (OUT) fraction of dried sample. This might be a result of excessive extraction and release of flavonoids via digestive enzyme application during the intestinal phase [36]. Moreover, jam processing was also found to increase the recovery of TF contents as compared to the fresh sample (Table 3). Therefore, these results may emphasize the positive effect of heating on the bioaccessibility of flavonoids.

After *in vitro* gastrointestinal digestion, TAC of fresh and processed red beetroot products were analyzed using DPPH, ABTS, FRAP, and CUPRAC assays as shown in Table 4. TAC values in IN and OUT fractions of samples could not be determined by the DPPH assay. Higher recovery values were measured with ABTS assay (17%–24%); whereas the differences in the recovery values of samples were not significant for this assay and also for the CUPRAC assay. According to the FRAP assay, pickled red beetroot had the lowest (4%) and the jam-processed red beetroot had the highest (11%) recovery values compared to others. In another study, according to the FRAP assay results, beetroot shot represented a 2.5-fold higher TAC following the *in vitro* digestion procedure as compared to its non-digested counterpart [37].

For the applied methods (except for DPPH), higher TAC values were obtained for the IN fractions of fresh, boiled, dried, jam, and pureed samples. For boiled and jam-processed red beetroot samples, higher TAC values were also determined in non-dialyzable (OUT) fractions. Jam processing led to a two-fold increased recovery in TAC values compared to the fresh red beetroots measured using CUPRAC assay (Table 4).

When we consider the pH conditions of TAC assays performed in this study, it can be hypothesized that the FRAP test (conducted at pH = 3.6) could be more appropriate to assess TAC in the PG samples, rather than IN samples. On the contrary, the ABTS assay (conducted at a pH = 8) could be more suitable to evaluate the IN samples with a pH of 7 [40]. Moreover, underestimation of TAC values can be explained that the variations in the structure of antioxidants after digestion may influence their reactivity with the generation of nitrogen radicals, biologically not quite viable in the DPPH assay [12]. Consequently, using a single assay may not evaluate the whole system that may affect the results. Therefore, an accurate view of the TAC of a foodstuff might be obtained using a variety of assays with different mechanisms [41].

## 3. Materials and Methods

### 3.1. Red Beetroot Products

Fresh red beetroot samples were collected in triplicates from a local market in Istanbul, Turkey. Home-processed red beetroot samples, including boiled, oven-dried, pickled, pureed, juice-processed, and jam-processed red beetroot were prepared using traditional methods as presented in Figure 1. All samples were ground to a fine powder using a precooled grinder (IKA Model A10) with liquid nitrogen and stored at −80 °C until analysis.

### 3.2. Extract Preparation

Prior to analysis, three independent extractions for each sample were performed using acidified (0.1% formic acid) aqueous-methanol (75%) extraction solvent, as described before by Capanoglu *et al.* [42]. Five grams of each grounded sample was extracted with 5 mL of extraction solvent and sonicated in an ultrasonic bath (Azakli, Istanbul, Turkey) for 15 min, which was then centrifuged (Hettich Zentrifugen Universal 32R, Hettich Zentrifugen, Tuttlingen, Germany) for 10 min at 4000 rpm at 5 °C and the supernatant was collected. Another 5 mL of extraction solvent was added to the pellet and the extraction procedure was repeated. Two supernatants were combined and stored at −20 °C until analysis.

### 3.3. Moisture Content

Moisture content of the fresh and processed red beetroot samples were measured with an automated infrared moisture content analyzer at 120 °C (MOC63u; Shimadzu Co., Tokyo, Japan).

### 3.4. Total Phenolic Content Determination

Total phenolic (TP) content of samples was determined using Folin-Ciocalteau reagent, according to the method modified from Singh *et al.* [43]. 200 µL sample extract was added to 1 mL freshly prepared Folin-Ciocalteau reagent (1:10, *v*/*v* with MQ water). Subsequently, 0.8 mL 7.5% sodium carbonate solution was added to the mixture. After 30 min of incubation at room temperature, absorbance was read at 765 nm, using a UV-Vis spectrophotometer (Shimadzu UV-1700; Shimadzu Corporation, Kyoto, Japan). Results were given as mg gallic acid equivalents (GAE) per 100 g fresh weight sample.

### 3.5. Total Flavonoid Content Determination

Total flavonoid (TF) content was determined based on the method described by Čanadanović-Brunet, *et al.* [44]. 1 mL sample extract was mixed with 4 mL distilled water and 300 µL 5% NaNO_2_ solution. After 5 min, 300 µL of 10% AlCl_3_ was added, and after an additional 6 min of incubation, 2 mL of 1 M NaOH was added to the mixture. The final volume was made up to 10 mL with MQ water. The absorbance was measured at 510 nm and the TF contents of extracts were given as rutin equivalents (RE) per 100 g fresh weight sample.

### 3.6. Total Antioxidant Capacity Determination

Total antioxidant capacities (TAC) of sample extracts were measured by using four different assays, including ABTS (2,2-azinobis (3-ethylbenzothiazoline-6-sulfonic acid) diammonium salt), DPPH (1,1-diphenyl-2-picrylhydrazyl), FRAP (ferric reducing antioxidant power), and CUPRAC (cupric ion reducing antioxidant capacity) methods. In all assays, Trolox was used as the authentic standard and the results were expressed in terms of milligrams of Trolox equivalent antioxidant capacity (TEAC) per 100 g fresh weight sample.

#### 3.6.1. ABTS (2,2-Azinobis (3-ethylbenzothiazoline-6-sulfonic acid) diammonium salt) Method

The ABTS assay was performed according to the method used by Miller and Rice-Evans [45], with some modifications. The stock solution was prepared by mixing ABTS (2.13 mM) and potassium persulfate (70.3 mM) solutions (100:1 *v*/*v*, respectively) and kept overnight at room temperature in the dark. ABTS stock solution was diluted in 50 mM potassium phosphate buffer (pH 8.0) in order to obtain the ABTS reaction solution, with an absorbance value of 0.90 (±0.20) at 734 nm. For the analysis, 1 mL ABTS reaction solution was added to 100 µL sample extract, and the absorbance was measured at 734 nm immediately after 1 min of initial mixing.

#### 3.6.2. DPPH (1,1-Diphenyl-2-picrylhydrazyl) Method

The DPPH assay was performed as described by Ravichandran *et al.* [29]. For the analysis, 6 mL of DPPH stock solution (1 × 10^−3^ M) was diluted in 100 mL of 75% methanol in order to obtain the DPPH reaction solution. Subsequently, 100 µL of each sample extract was mixed with 3.9 mL of DPPH reaction solution. After a 30 min incubation period, the absorbance of the mixture was measured at 515 nm.

#### 3.6.3. FRAP (Ferric Reducing Antioxidant Power) Method

The FRAP assay was performed according to the procedure of Benzie and Strain [46]. FRAP reagent was prepared from a blend of acetate buffer (pH 3.6), 10 mM TPTZ (2,4,6-tris(2-pyridyl)-*s*-triazine) solution and 20 mM ferric chloride in ratios of 10:1:1 (*v*/*v*/*v*), respectively. 900 µL of freshly prepared FRAP reagent was mixed with 100 µL sample extract. After 4 min of incubation time, the absorbance of the reaction mixture was recorded at 593 nm.

#### 3.6.4. CUPRAC (Cupric Ion Reducing Antioxidant Capacity) Method

In this study, the CUPRAC assay developed by Apak *et al.* [47] was performed. 1 mL of 10 mM CuCl_2_, 1 mL of 7.5 mM neocuproine, and 1 mL of 1 M NH_4_Ac (pH: 7) was added to 100 µL of sample extract. Subsequently, 1 mL of MQ water was added to this mixture in order to have the final volume of 4.1 mL. After 30 min of incubation at room temperature, absorbance was read at 450 nm.

### 3.7. HPLC (High-Pressure Liquid Chromatography) Analysis of Betanin

Red beetroot sample extracts were filtered through a 0.45 µm membrane filter and were injected to the Waters 2695 HPLC system (Waters, Milford, MA, USA) with a PDA (Waters 2996) detector, in order for the identification and quantification of individual red beetroot antioxidants. Stationary phase was Supercosil^®^ (Sigma-Aldrich, St. Louis, MO, USA) LC-18 column (25 × 4.6 mm, 5 µm) and the mobile phase A was Milli-Q water with 0.1% (*v*/*v*) trifluoroacetic acid (TFA), while mobile phase B was acetonitrile with 0.1% (*v*/*v*) TFA. The flow rate was set as 1 mL/min. A linear gradient was 95% solvent A and 5% solvent B from 0 to 45 min; 65% solvent A and 35% solvent B from 45 to 47 min; 25% solvent A and 75% solvent B from 47 to 54 min; and at 54 min returned to the initial conditions [48]. Eluting compounds were monitored continuously between 240 and 530 nm using a Waters 996 Photo Diode Array (PDA) detector. Betanin standard (Sigma-Aldrich, CDS000584) was used to compare absorbance spectra and retention times of eluting peaks. For quantification, calibration curves of available pure standards (0–80 µg/mL) were used as reference.

### 3.8. In Vitro Gastrointestinal Digestion

**Figure 4:**
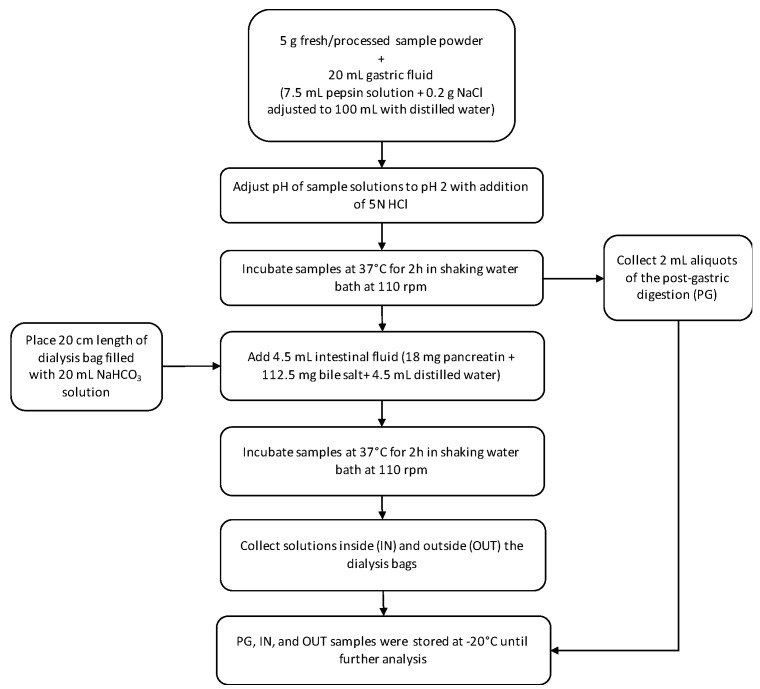
Illustration of the flow chart for the *in vitro* gastrointestinal digestion procedure.

*In vitro* gastric and intestinal digestion phases were mimicked, according to the method described before by McDougall *et al.* [49], to determine the variation in the bioaccessibility of red beetroot bioactives during processing. The mimicked digestion procedure was presented in Figure 4. A blank sample was prepared with the same chemicals, and without sample. All samples (IN, OUT, PG, and blank) were stored at −20 °C until analysis. Before analysis, the samples were thawed and centrifuged at 23,000× *g* (Universal 32R; Hettich Zentrifugen) and then, assayed for TP, TF, and TAC, using the methods described above.

### 3.9. Statistical Analysis

Data were collected from three independent extractions and reported as mean ± standard deviation. The correlation coefficients (*R*^2^) for spectrophotometric assays, overall mean, and standard error values were calculated by using the Microsoft Office Excel 2011 software (Microsoft Corporation, Redmond, WA, USA). One-way analysis of variance (ANOVA) followed by the Duncan *post hoc* test were used to compare the treatments (*p* < 0.05).

## 4. Conclusions

In conclusion, the importance of this study was to evaluate the effects of various home-processing techniques on total amount and capacity of red beetroot antioxidants, as well as on the fate of red beetroot bioactives during *in vitro* gastrointestinal digestion. Our results revealed that heat application might have a protecting or an enhancing effect on TP and TF contents, and TAC of red beetroot, depending on the values obtained for dried and pureed samples. In addition, dried red beetroot represented a higher recovery for total TF contents following the *in vitro* digestion procedure applied compared to the recovery of TF in the other fresh and processed red beetroot samples. Furthermore, jam-processing provided higher recoveries for TP and TF contents, as well as for TAC (determined via CUPRAC assay) values after *in vitro* gastrointestinal digestion compared to the other digested red beetroot samples. It is important to carry out more studies with various antioxidant-rich food materials, by using their fresh and processed forms, in order to support and improve the knowledge on the potential availability of these bioactives in different food matrices, specifically after digestion, since the metabolism of food components in the gastrointestinal tract determines their bioactivity and effects on health. Antioxidant potential of foods prior to digestion can be used for comparisons, however this may not reflect the potential health effects. Therefore, further studies are necessary to elucidate the fate of antioxidants during the digestion process which determines their release from the food matrix (bioaccessibility) and their functioning throughout the metabolism.

## Figures and Tables

**Table 1:** The effect of different home-processing methods on red beetroot antioxidants according to the spectrophotometric analyses performed *.

Samples	Total Phenolic Content (TP)	Total Flavonoid Content (TF)	Total Antioxidant Capacity (TAC)			
			DPPH ^‡^	ABTS ^‡^	FRAP ^‡^	CUPRAC ^‡^
Fresh red beetroot	255 ± 48 ^b^	260 ± 13 ^a,b^	137 ± 11 ^a,b^	190 ± 12 ^a^	181 ± 8 ^a^	3889 ± 82 ^a^
Boiled red beetroot	238 ± 15 ^b^	261 ± 38 ^a,b^	131 ± 6 ^a,b^	158 ± 9 ^b^	126 ± 17 ^c,d^	3376 ± 377 ^b^
Dried red beetroot	347 ± 24 ^a^	230 ± 53 ^b^	143 ± 8 ^a^	188 ± 0.4 ^a^	170 ± 25 ^a,b^	3567 ± 307 ^a,b^
Pickled red beetroot	192 ± 50 ^c^	173 ± 38 ^c^	114 ± 15 ^c^	122 ± 29 ^c^	66 ± 5 ^e^	2413 ± 425 ^d^
Red beetroot jam	231 ± 36 ^b,c^	143 ± 37 ^c^	127 ± 15 ^b^	160 ± 26 ^b^	126 ± 31 ^c,d^	2931 ± 528 ^c^
Red beetroot juice	225 ± 15 ^b,c^	126 ± 14 ^c^	110 ± 9 ^c^	126 ± 12 ^c^	112 ± 16 ^d^	2397 ± 195 ^d^
Red beetroot puree	236 ± 18 ^b^	290± 53 ^a^	139 ± 4 ^a,b^	186 ± 11 ^a^	148 ± 24 ^b,c^	3529 ± 243 ^a,b^

* Data represent average values ± standard deviation of three independent extractions from each sample on a wet basis. Different letters in the columns represent statistically significant differences (*p* < 0.05). TP results were given as mg gallic acid equivalent (GAE)/100 g sample; TF Contents of extracts were given as mg rutin equivalent (RE)/100 g sample; TAC results were given as mg trolox equivalent antioxidant capacity (TEAC)/100 g sample; ^‡^, DPPH (1,1-diphenyl-2-picrylhydrazyl), CUPRAC (Cupric Ion Reducing Antioxidant Capacity), ABTS ((2,2-azinobis (3-ethylbenzothiazoline-6-sulfonic acid) diammonium salt), and FRAP (Ferric Reducing Antioxidant Power).

**Table 2:** Changes in the total phenolic contents (TP; mg GAE 100 g^−1^ sample, wet basis) of fresh and processed red beetroot products during *in vitro* gastrointestinal digestion *.

Sample Names	Initial ^‡^	PG ^‡^	IN ^‡^	OUT ^‡^	Recovery (%) ^‡^
Fresh red beetroot	255 ± 48 ^b^	55 ± 7 ^b,c^	17 ± 2 ^b^	37 ± 5 ^b^	7 ± 2 ^b^
Boiled red beetroot	238 ± 15 ^b^	61 ± 6 ^b^	18 ± 4 ^b^	34 ± 6 ^b^	8 ± 0.6 ^b^
Dried red beetroot	347 ± 23 ^a^	43 ± 8 ^c,d^	19 ± 4 ^b^	37 ± 7 ^b^	5 ± 0.8 ^b,c^
Pickled red beetroot	192± 50 ^c^	29 ± 6 ^e^	8 ± 1 ^d^	16 ± 1 ^c^	4 ± 1 ^d^
Red beetroot jam	231 ± 36 ^b,c^	75 ± 19 ^a^	37 ± 3 ^a^	59 ± 15 ^a^	16 ± 2 ^a^
Red beetroot juice	225 ± 15 ^b,c^	35 ± 8 ^d,e^	12 ± 2 ^c^	13 ± 1 ^c^	5 ± 0.6 ^b,c^
Red beetroot puree	236 ± 18 ^b^	55 ± 5 ^b,c^	13 ± 2 ^c^	30 ± 6 ^b^	6 ± 1 ^b,c^

* Data represent average values ± standard deviation of three independent extractions from each sample. Different letters in the columns represent statistically significant differences (*p* < 0.05); ^‡^, the terms represent: Initial, as initially determined after extraction procedure; PG, after gastric digestion; IN, dialyzed fraction after intestinal digestion; OUT, non-dialyzed fraction after intestinal digestion; Recovery, values obtained in the IN fraction/values obtained in the initial sample as percentage.

**Table 3:** Changes in the total flavonoid contents (TF; mg RE 100 g^−1^ sample, wet basis) of fresh and processed red beetroot products during *in vitro* gastrointestinal digestion *.

Sample Names	Initial ^‡^	PG ^‡^	IN ^‡^	OUT ^‡^	Recovery (%) ^‡^
Fresh Red Beetroot	260 ± 13 ^a,b^	105 ± 5 ^d^	33 ± 19 ^b^	100 ± 26 ^b^	13 ± 5 ^c^
Boiled Red Beetroot	261 ± 38 ^a,b^	148 ± 17 ^c^	35 ± 1 ^b^	82 ± 12 ^b^	13 ± 2 ^b^
Dried Red Beetroot	230 ± 53 ^b^	288 ± 39 ^a^	93 ± 35 ^a^	255 ± 57 ^a^	34 ± 8 ^a^
Pickled Red Beetroot	173 ± 38 ^c^	84 ± 17 ^d,e^	29 ± 26 ^b^	26 ± 9 ^c^	10 ± 3 ^c^
Red Beetroot Jam	143 ± 37 ^c^	188 ± 20 ^b^	31 ± 4 ^b^	78 ± 11 ^b^	23 ± 5 ^b^
Red Beetroot Juice	126 ± 14 ^c^	65 ± 8 ^e^	17 ± 9 ^b^	72 ± 56 ^b,c^	10 ± 0.7 ^c^
Red Beetroot Puree	290 ± 53 ^a^	170 ± 26 ^b,c^	18 ± 4 ^b^	73 ± 20 ^b,c^	7 ± 2 ^c^

* Data represent average values ± standard deviation of three independent extractions from each sample. Different letters in the columns represent statistically significant differences (*p* < 0.05); ^‡^, the terms represent: Initial, as initially determined after extraction procedure; PG, after gastric digestion; IN, dialyzed fraction after intestinal digestion; OUT, non-dialyzed fraction after intestinal digestion; Recovery, values obtained in the IN fraction/values obtained in the initial sample as percentage.

**Table 4:** Changes in the total antioxidant capacity (TAC; mg TEAC 100 g^−1^ sample, wet basis) values of fresh and processed red beetroot products, determined using four *in vitro* tests during *in vitro* gastrointestinal digestion *.

Assay	Samples	Initial ^‡^	PG ^‡^	IN ^‡^	OUT ^‡^	Recovery (%) ^‡^
DPPH	Fresh red beetroot	137 ± 11 ^a,b^	83 ± 12 ^a,b^	nd ^†^	nd ^†^	nd ^†^
	Boiled red beetroot	131 ± 6 ^a,b^	72.3 ± 4 ^b^	nd ^†^	nd ^†^	nd ^†^
	Dried red beetroot	143 ± 8 ^a^	76 ± 10 ^b^	nd ^†^	nd ^†^	nd ^†^
	Pickled red beetroot	114 ± 15 ^c^	44 ± 11 ^c^	nd ^†^	nd ^†^	nd ^†^
	Red beetroot jam	127 ± 15 ^b^	91 ± 8 ^a^	nd ^†^	nd ^†^	nd ^†^
	Red beetroot juice	110 ± 9 ^c^	43 ± 11 ^c^	nd ^†^	nd ^†^	nd ^†^
	Red beetroot puree	139 ± 4 ^a,b^	78 ± 5 ^b^	nd ^†^	nd ^†^	nd ^†^
CUPRAC	Fresh red beetroot	3889 ± 82 ^a^	209 ± 32 ^a^	18 ± 7 ^b^	96 ± 45 ^a,b,c^	0.5 ± 0.2 ^b^
	Boiled red beetroot	3375 ± 377 ^b^	216 ± 28 ^a^	15 ± 16 ^b^	48 ± 23 ^d,e^	0.4 ± 0.2 ^b^
	Dried red beetroot	3567 ± 307 ^a,b^	231 ± 55 ^a^	15 ± 5 ^b^	121 ± 46 ^a^	0.4 ± 0.1 ^b^
	Pickled red beetroot	2413± 425 ^d^	111 ± 23 ^b^	nd ^†,c^	12 ± 5 ^e^	nd ^†^
	Red beetroot jam	2931 ± 528 ^c^	246 ± 20 ^a^	39 ± 11 ^a^	108 ± 19 ^a,b^	1.4 ± 0.6 ^a^
	Red beetroot juice	2397 ± 195 ^d^	139 ± 14 ^b^	9 ± 3 ^b,c^	57 ± 30 ^c,d^	0.4 ± 0.1 ^b^
	Red beetroot puree	3529 ± 243 ^a,b^	247 ± 34 ^a^	18 ± 13 ^b,c^	74 ± 7 ^b,c,d^	0.2 ± 0.01 ^b^
ABTS	Fresh red beetroot	189 ± 12 ^a^	30 ± 0.0 ^b^	33 ± 0.1 ^a^	36 ± 0.4 ^a,^^b^	17 ± 6 ^a^
	Boiled red beetroot	158 ± 9 ^b^	30 ± 0.0 ^b^	33 ± 0.3 ^a^	37 ± 0.2 ^a^	21 ± 1 ^a^
	Dried red beetroot	188 ± 0.4 ^a^	92 ± 71 ^a^	33 ± 0.2 ^a^	36 ± 0.6 ^b^	18 ± 0.1 ^a^
	Pickled red beetroot	122 ± 29 ^c^	28 ± 0.0 ^b^	25 ± 5 ^c^	32 ± 1.4 ^d^	21 ± 5 ^a^
	Red beetroot jam	160 ± 26 ^b^	30 ± 0.0 ^b^	33 ± 0.0 ^a^	37 ± 0.1 ^a^	21 ± 4 ^a^
	Red beetroot juice	126 ± 12 ^c^	28 ± 0.0 ^b^	30 ± 1.0 ^b^	33 ± 1.0 ^c^	24 ± 2 ^a^
	Red beetroot puree	186 ± 11 ^a^	30 ± 0.1 ^b^	33 ± 0.0 ^a^	36 ± 0.4 ^a,b^	18 ± 0.8 ^a^
FRAP	Fresh red beetroot	181± 8 ^a^	50 ± 0.4 ^b^	19 ± 5 ^a^	38 ± 4 ^a^	10 ± 3 ^a^
	Boiled red beetroot	126 ± 17 ^c,d^	50 ± 0.4 ^b^	11 ± 3 ^c,d^	25 ± 2 ^c^	9 ± 2 ^a^
	Dried red beetroot	170 ± 25 ^a,b^	51 ± 0.3 ^a^	17 ± 6 ^a,b^	36 ± 9 ^a^	10 ± 4 ^a^
	Pickled red beetroot	66 ± 5 ^e^	48 ± 0.5^c^	1 ± 1 ^e^	9 ± 0.6 ^e^	3 ± 0.3 ^b^
	Red beetroot jam	126 ± 31 ^c,d^	48 ± 0.3 ^c^	14 ± 0.7 ^b,c^	33 ± 2 ^a,b^	11 ± 3 ^a^
	Red beetroot juice	112 ± 16 ^d^	51 ± 0.6 ^b^	9 ± 1.6 ^d^	19 ± 4 ^d^	8 ± 2 ^a^
	Red beetroot puree	148 ± 24 ^b,c^	50 ± 0.3 ^b^	12 ± 1 ^c,d^	29 ± 2 ^b,c^	8 ± 1 ^a^

* Data represent average values ± standard deviation of three independent extractions from each sample. Different letters in the columns represent statistically significant differences (*p* < 0.05); ^†^ nd, not determined; ^‡^, the terms represent: Initial, as initially determined after extraction procedure; PG, after gastric digestion; IN, dialyzed fraction after intestinal digestion; OUT, non-dialyzed fraction after intestinal digestion; Recovery, values obtained in the IN fraction/values obtained in the initial sample as percentage.
